# Supplementary material for: Are carbon emissions trading and green financial instruments synergistic? -Comprehensive quantitative research based on content analysis
Source: PLoS One. 2024 Mar 7;19(3):e0298601. doi: 10.1371/journal.pone.0298601 (PMC10919601; doi:10.1371/journal.pone.0298601)
Supplement: S1 Data — (ZIP) [file pone.0298601.s001.zip › Data/地级市Z_F工作报告.zip!/║┌┴·╜¡/╣■╢√▒⌡╩╨/└┤╫╘╣1⁄2╓┌║┼í╛┬φ┐╦╩2╛▌═°í┐_2013.docx]

各位代表：

　　现在，我代表市人民政府向大会报告工作，请予审议，并请市政协各位委员提出意见。

　　一、2012年工作简要回顾

　　过去一年，是新一届政府开局之年。面对严峻的国际经济形势和繁重的改革发展稳定任务，我们在省委、省政府和市委的正确领导下，紧紧依靠全市人民，以迎接和学习贯彻党的十八大精神为契机，按照省委“八大经济区”、“十大工程”的战略部署，牢牢把握科学发展主题和稳中求进总基调，坚定实施新战略，奋力推进新跨越，攻坚克难、拼搏进取、奋力前行，全市经济社会发展迈出了坚实步伐。

　　经济保持平稳增长。预计地区生产总值比上年增长10%左右。实现固定资产投资3915亿元，增长30%。社会消费品零售总额增长15%左右。完成公共财政预算总收入581.4亿元，其中地方公共财政预算收入354.7亿元，分别增长15.8%和18.1%。万元GDP综合能耗下降3.6%以上。

　　产业结构加快调整。工业转型升级步伐加快，通过高新技术改造提升传统优势产业取得积极进展，战略性新兴产业不断培育壮大，食品工业成为我市首个超千亿元产业。农业结构进一步优化，新增水田80万亩，绿色有机产品认证面积稳定在3200万亩以上，畜牧业占农林牧渔业总产值比重达46.5%左右。服务业加快提档升级，旅游总收入达553亿元，增长20.5%，成功打造了“迷人的哈尔滨之夏”城市品牌，在中国十佳避暑旅游城市评比中名列第二位；金融机构人民币存贷款余额分别突破7000亿元和5000亿元大关；物流、会展、总部和楼宇经济等新兴服务业呈现良好发展态势。

　　各项改革继续推进。国企改革不断深化，厂办大集体改制基本完成。国有资产战略性重组步伐加快，市政集团正式挂牌运营，交通集团、文旅集团组建全面启动，市属企业国有权益总额达2509亿元，增长30%。医药卫生体制改革扎实推进，新农合实现全覆盖，基层医疗卫生机构实施基本药物制度，10所县（市）医院完成升级改造，群众看病负担减轻，就医条件得到改善。农村产权制度、财政金融体制、事业单位、文化体制等领域改革取得积极进展。

　　人民生活持续改善。70个惠民项目全部落实，完成投资292亿元，超年计划54亿元。城市居民人均可支配收入、农村居民人均纯收入分别达22500元和11443元，增长12.3%和19.1%。各级各类教育均衡发展，财政教育支出达118.6亿元，增长30.2%，幼儿师范高等专科学校新校区、哈尔滨职业教育园建设顺利推进。科技创新步伐加快，被评为全国首批知识产权示范城市。食品药品安全管理工作得到加强。人口自然增长率为2.1％。。文化体育事业蓬勃发展，乡（镇）文化站和农家书屋提前两年实现全覆盖，在第十二届全国冬运会上取得历史最好成绩。社会管理“强基工程”全面启动，新建千米以上中心社区30个，涌现出了道里、通河、延寿、五常等一批创新社区管理的先进典型。

　　精神文明与民主法治建设得到加强。文明城市创建工作深入推进，在全国城市文明程度指数测评中列提名城市第三位。思想道德建设成效明显，涌现出以谢尚威为代表的一批在全社会引起强烈反响的道德楷模，形成了“哈尔滨风尚”。自觉接受人大法律监督和政协民主监督，人大代表建议、政协提案办复率均达100%。提请市人大常委会审议地方性法规3项，制定政府规章9项。“六五”普法扎实推进。政府处置突发公共事件的能力明显增强，成功应对“布拉万”台风灾害，及时妥善处置“8·14”辽阳街路面塌陷事故、“8·24”道路交通事故以及由此引发的社会舆论事件。大安全社会治安工作格局基本形成，刑事案件发案总量在副省级城市中最低，重特大公共安全事故大幅下降，公众安全感满意度达94.6%。信访积案化解率达80%。

　　各位代表，过去一年，我们按照市第十三次党代会和市十四届人大一次会议的新要求、新部署，咬定青山不放松，持之以恒抓推进，围绕全局促落实，为推动更好更快发展奠定了坚实基础、提供了重要保障。

　　一是坚持突出产业发展核心地位，进一步夯实打牢加快发展根基。把产业建设作为实施新战略、实现新跨越的关键点和突破口，强基固本，合力推进，产业发展基础更加坚实。突出规划引领。依据资源禀赋和区域经济比较优势，组织编制全市产业布局规划，明晰园区定位和区县（市）主导产业发展方向，出台项目异地流转政策，形成了优势互补、错位发展、统筹推进的产业建设新格局。强化创新驱动。把立足点放在科技创新与产业建设加速融合上，投入3000万元，联手哈工大等大学大所，创建了科技成果转化平台，集聚重大科技成果转化及产业化项目600个，实现落地转化110个，开辟了科技创新成果本地转化的新途径。抓好园区建设。投入3800万元，开展园区规划环境影响评价，设立3亿元引导性资金，支持园区基础设施建设，园区产业承载力、吸引力和集聚力进一步增强。经开区新引进超百亿项目3个、世界500强企业7户，高新区竣工投产研发和产业化项目50个，哈药总厂等23家知名医药企业落户利民开发区，宾西开发区成为东北地区最大的现代包装产业生产基地，五常牛家等县域工业园展示了快速发展的新形象。扩大招商引资。把招商作为推动产业建设发展的着力点，主动出击、强力推进，新签约的128个项目中产业项目达104个，当年开工69个，到位资金167.5亿元。全市实际使用外资18.9亿美元，增长18%；国内经济技术合作到位资金808亿元，增长20%。完善金融体系。着力强化金融对产业发展的支撑作用，全年新增贷款900亿元，发行企业债券12亿元，新组建的企业信用融资担保服务中心提供担保资金达105.5亿元。汇丰银行正式运营，法兴银行获批筹建，博实股份成功上市。创优发展环境。进一步加大政府自身建设力度，开展了以行政问责为核心的“政风建设年”活动，严肃查处破坏经济发展环境相关案件225起，问责公职人员399人，极大地教育和警示了广大公务员，政府的依法行政能力和服务效率得到较大提高。通过一年来的创新实践，可以说，抓产业的思路更清晰了、导向更明确了、措施更有效了。只要我们坚定不移地抓推进、促落实，哈尔滨就一定会在科学发展新跨越的道路上阔步前行！

　　二是坚持增强经济发展后劲，进一步打造科学发展新优势。围绕构建“4+6+1”产业体系，强力推进产业大项目建设，培育壮大新的经济增长点。突出抓好省市重点产业项目建设。省市重点产业项目完成年度投资588亿元，其中154个省重点产业项目完成投资350亿元左右。东轻超大规格铝板带材等36个亿元以上项目相继竣工投产，成为推动经济加快发展的新生力量。全力抓工业、遏下滑、保增长，综合施策、协力推进，工业经济实现企稳回升、逐季走高。切实加大文化产业项目建设力度。大剧院、音乐厅等标志性文化大项目及中华巴洛克二期等改造项目按计划推进，关东古巷等一批文化设施开业运营，平房动漫基地成为全国文化与科技融合示范基地。努力增强物流、会展业项目拉动作用。润恒一期、商德金属物流等一批大型物流项目陆续建成使用，现代物流格局初步形成；“哈尔滨世界农业博览会”成功举办，使我市的国际知名度和美誉度得到了进一步提升。

　　三是坚持实施富民强县战略，进一步推动县域经济更好更快发展。着力推进农业现代化、县域工业化、农村城镇化、区域一体化，县域经济发展活力不断增强，实现县域生产总值增长12%，完成地方公共财政预算收入52.5亿元，增长30.9%。大力推进农牧业生产，农业综合生产能力显著提高。粮食生产战胜多种自然灾害再夺大丰收，产量由上年的284.6亿斤提高到320亿斤，被国务院授予“全国粮食生产先进市”称号；畜牧养殖专业带（区）初步形成，肉、蛋、奶总量达274.8万吨。大力推进产业强县工程，县域工业实力进一步增强。通过规划引领、拉练推动、政策扶持，加快县域产业项目建设，新建续建宾西食品、延大牧业等大项目192个，木兰、通河、方正、延寿结束了没有亿元大项目的历史。大力推进新农村建设，城镇化进程持续加快。撤并7个乡（镇）、137个村（屯），建设市级新农村示范村20个，完成扶贫开发整村推进68个，开工建设783公里农村公路，交工通车611公里，改造危险桥梁273座，依兰松花江大桥主体合龙，农村面貌发生了许多令人振奋的新变化。

　　四是坚持高标准规划建设管理，进一步提升城市环境质量和形象品位。围绕建设现代化国际化大城市目标，修改城市总体规划，强化规划管理，旧城核心区域内除政府主导的优化城市功能、提升城市空间品质及棚改项目外，住宅项目一律停批，进一步彰显大气、洋气、神气城市风格。着力完善城市交通体系，努力让群众出行更方便。新建改造各类道路146条、桥涵34座，学府立交桥、王岗高架桥、八区和保健路地道桥建成通车，公滨路、延兴路、化工路、松浦大道全线贯通，哈西群力联络空间区域改造成效突出，地铁1号线通车试运行，哈西客站建成使用，哈大高铁正式开通，城市立体化交通网络日趋完善；新建停车场34个，新增停车泊位3.45万个，新增、更新大容量公交车600台，23座新型公交场站投入使用,成为“公交都市”全国首批创建单位。着力加强源网设施建设，努力让市民不再为身边琐事而担忧。改造水、电、气、热管网2147公里，拆并小锅炉505台，新增供热能力2600万平方米，维修老旧住宅1000万平方米，城市运行保障水平得到较大提升。着力推进综合整治，努力让百姓生活环境更优美。牌匾广告、临街踏步、道路检查井等专项整治成效明显，违法“小广告”得到基本遏制；实施既有建筑节能改造410万平方米，友谊路、经纬街成为城市节能改造的样板；大力推进森林城市建设，新增绿地250公顷，完成造林13.7万亩；积极修复水生态系统，“三沟”结束了污水直排松花江的历史，阿什河下游截污工程进展顺利，松花江水质主要污染指标下降3.7%；北国水城一期初见成效。着力完善城市管理体制和长效机制，努力让百姓门前更干净。明确职责定位，下放管理权限，强化各区和街道办事处的主体作用，增加环卫投入，新招大专学历以上环卫工人448人，城区清运垃圾120万吨，无害化处理率达85%，污水集中处理率达83%，机械化作业、数字化管控、常态化管理水平明显提升，城市环境面貌和干净程度明显好于往年。着力拆除违章建筑，努力让百姓生活的城市更规范。累计拆除违章建筑1万余处、371万平方米，拆违不仅顺应了民心，更提高了党和政府的威信，维护了社会公平正义。

　　五是坚持为群众办实事办好事，进一步增进全市人民福祉。把保障和改善民生作为政府工作的全部出发点和落脚点，努力让全市人民更多地分享改革发展成果。立足居者有其屋，开工建设保障房3.9万套，竣工51557套，为1.6万户群众提供廉租房和经济适用房补贴，超额完成国家和省下达目标。道外区桥头屯棚改走出了一条全市统筹、多点安置、快速推进、和谐搬迁、群众满意的新路子。公积金支持保障房建设资金达15亿元。改造农村泥草房6.2万户、540万平方米。立足创业有舞台、就业有岗位，持续推动创业带动就业，城镇新增就业12.6万人，登记失业率控制在3.4%以内，转移农村劳动力138.3万人，成为全国创业先进城市。立足提高社会保障能力，养老保险政策实现全覆盖，企业退休人员基本养老金月均增加206元；城镇职工、城镇居民基本医疗保险年度最高支付限额提高到25万元和11万元；城乡最低生活保障标准提高到每月410元和每年1718元；为35万户困难群众发放社会救助金11亿元；新改建社会养老机构47家。西泉眼水库返迁移民安置问题得到彻底解决。经过一年的努力，各项惠民承诺全部兑现，百姓生活水平和质量得到较大提高。

　　各位代表，过去的一年，是哈尔滨经济社会发展历程中极不平凡的一年，是直面矛盾、破解难题，经济社会发展迈上转型振兴新台阶的一年，是抢抓机遇、苦干实干，科学发展新跨越基础不断夯实的一年。这些成绩的取得，是省委、省政府和市委正确领导的结果，是全市人民团结奋斗的结果。在此，我代表市人民政府，向所有关心支持哈尔滨事业发展的同志们、朋友们，致以崇高的敬意和衷心的感谢！

　　一年来的发展实践使我们深切地感到：之所以能够在发展中创新突破、在困难中奋力前行，关键是我们牢牢把握中央稳中求进总基调不动摇，坚持贯彻落实省委、省政府和市委战略部署不懈怠，各级领导班子精诚团结、锐意进取，人大代表、政协委员及中省直单位和社会各界鼎力支持、全力配合，开创了万众同心、开拓进取、狠抓落实的新局面；关键是我们面对危机不言弃、面对困难不低头，坚持用发展的办法解决前进中的问题，举全市之力、集全民之智，抓产业、上项目，调结构、转方式，惠民生、促和谐，创造了顽强拼搏、奋发有为、加快发展的新业绩；关键是我们不等不靠、因势利导，敏锐地发现机遇，奋力地抢抓机遇，在争取政策、项目、资金等方面取得重大进展，强化了大干快上、加快崛起、再创辉煌的新支撑；关键是我们始终坚持全心全意为人民服务宗旨，让发展成果最大限度地惠及百姓，充分调动了全市人民关心发展、支持发展、参与发展的积极性和创造性，增强了埋头苦干、奋勇争先、干事创业的新动力。这些在实践中积累的宝贵财富，需要我们倍加重视、倍加珍惜、倍加爱护。只要持之以恒地坚持、与时俱进地创新，发展就会见实效，事业就会有成就，人民就会增福祉。

　　在肯定成绩的同时，我们也要清醒地认识到发展中还存在不少困难和问题：发展速度不快、产业结构不优、体制机制障碍等问题仍然十分突出，经济发展抵御市场风险的能力较弱，受世界经济复苏乏力、国内外市场需求萎缩、经济下行压力加大等因素的影响，全市地区生产总值、规上工业增加值、进出口总额指标未能实现年初预期，做大产业规模、提升发展质量、深化改革开放依然任重而道远；城市功能不完备，水电气热等源网配套设施严重滞后于城市快速发展需要，城市综合治理和应急管理水平有待于进一步提高；城乡居民收入依然较低，社会保障和基本公共服务还不到位，改善民生需要付出更艰辛的努力；政府职能和作风转变还有很大差距，部分公务员思想、作风、能力与科学发展要求不相适应的问题还依然存在，腐败现象和损害群众利益的问题还时有发生。对这些问题，我们要高度重视，认真剖析成因，探求破解之策，在克服困难中开拓前进，在不断创新中加快发展。

　　二、2013年主要任务

　　2013年是贯彻落实十八大精神的开局之年，也是实施“十二五”规划承前启后的一年，更是为我市全面建成小康社会奠定坚实基础的关键之年。做好今年工作责任重大、意义深远。

　　新的一年，哈尔滨加快发展机遇难得。虽然国际经济形势依然错综复杂、充满变数，世界经济低速增长态势仍将延续，但我国发展仍处于重要战略机遇期的基本判断没有变。面对新挑战、新机遇，党和国家因势利导、顺势而为，更加注重内需拉动，把推进城镇化作为经济结构调整的战略重点，有利于我们依托大城市、大农村，在加速城镇化、加快城乡一体化上迈出更大步伐；更加注重“四化”同步推进，有利于我们依托老工业基地奠定的产业基础，在加速经济结构调整、产业转型升级上取得更大进展；更加注重巩固农业基础和确保粮食安全，有利于我们依托国家重要商品粮基地和绿色食品基地，在发展现代农业、提升农业产业化和市场化程度上获得更大支持。省委、省政府在全力推进“八大经济区”、“十大工程”建设中，更加注重发挥省会龙头带动作用，有利于我们依托省市共建、政策支持，在率先发展、跨越赶超上实现更大突破。只要我们抢抓机遇，坚定信心，埋头苦干，哈尔滨就一定会乘势而上、奋勇争先！

　　新的一年，哈尔滨加快发展潜力巨大。我市农业、土地、电力等资源富集，江河、湿地、森林等生态优良，在资源和环境约束加剧的背景下，我市的资源和生态承载优势越来越明显。作为地处东北亚区域中心和国家沿边开发开放带上的特大型城市，在中俄全面加强战略协作伙伴关系、俄罗斯入世和远东大开发的背景下，对俄合作战略升级“桥头堡”和“枢纽站”的区位优势越来越明显。哈尔滨科技实力较强，在经济发展更多依靠科技进步的背景下，创新驱动发展的潜在优势越来越明显。作为全国老工业基地城市，在经济发展特别注重实体经济和改革红利的背景下，传统产业升级改造、战略性新兴产业发展的基础优势越来越明显。只要我们发挥好这些比较优势，最大限度地释放发展潜能，哈尔滨就一定会赢得主动、赢得未来！

　　新的一年，哈尔滨加快发展蓄势待发。实施新战略、实现科学发展新跨越，鼓舞人心，引领未来，催人奋进。随着产业发展和城市建设的深入推进，城市骨架进一步拉开，大哈尔滨格局初步形成，产城融合态势日趋显现，跨越赶超、加快发展的空间更加广阔；推进经济持续健康发展思路清晰、重点突出，万众一心抓产业、凝神聚力上项目，已成为全市上下的思想共识和自觉行动，跨越赶超、加快发展的方向更加明确；适应竞相发展新形势，顺应人民群众新期待，领先于全国全省实现全面小康目标，已成为广大干部群众的共同追求和坚定信念，跨越赶超、加快发展的劲头更加高涨。只要我们持之以恒，不动摇、不懈怠、不折腾，顽强拼搏、艰苦奋斗、不懈努力，哈尔滨就一定会谱写出产业大发展、城市大变化、民生大改善的新篇章！

　　按照市委十三届四次全会要求，政府工作的指导思想是：全面贯彻落实党的十八大精神和省委“八大经济区”、“十大工程”战略部署，继续坚定实施新战略，以产业发展为牵动，以改革开放为动力，着力推进产业项目建设和结构调整，着力促进城乡统筹和一体化发展，着力改善民生和保障人民民主权利，着力加快文化名城和文明城市建设，着力提高公共服务水平和创新社会管理，着力加强生态资源保护开发和循环利用，努力推动哈尔滨科学发展新跨越，为建设现代化国际化大城市奠定基础。

　　综合考虑各种因素，今年经济社会发展的主要预期目标是：地区生产总值增长12%左右；固定资产投资增长32%以上；规模以上工业增加值增长14%左右；社会消费品零售总额增长15%左右；地方公共财政预算收入增长15%左右；市属外贸进出口总额增长10%左右；城市居民人均可支配收入增长12%以上，农村居民人均纯收入增长14%以上；城镇居民登记失业率控制在4.3%以内；万元GDP综合能耗下降3.56%以上。

　　为实现上述思路和目标，着重做好以下六项工作：

　　（一）奋力推动产业大建设大发展。坚定不移地走产业立市、产业强市、产业兴市之路，把产业建设的着力点放在盘活存量调结构、做大增量促发展上，通过项目牵动、创新驱动、园区带动，真正使今年成为产业大引进、投资大增加、项目大开工的重要一年。一是坚决打赢产业项目建设攻坚战。全年实施省市重点产业项目240个，完成投资600亿元；谋划亿元以上项目500个、10亿元以上项目100个；产业项目投资占固定资产投资比重达35%以上。围绕改造提升传统产业，培育壮大战略性新兴产业，重点抓好百威英博等重大技改项目100个、中小型燃汽轮机等新兴产业项目100个，确保空客复合材料中心等50个项目竣工投产。新增规模以上工业企业80户。加快工业化与信息化融合，推动制造模式向数字化、网络化、智能化、服务化方向转变。围绕完善提高现代服务业，加快松花江避暑城、万达文化旅游城等标志性大项目建设，着力打造湿地景区，继续办好“迷人的哈尔滨之夏”文化旅游活动，进一步提升“冰城夏都”品牌，全年旅游总收入增长20%以上。加快华南城、义乌小商品城、双城雨润等商贸物流大项目建设，确保群力远大购物广场、新汇国际购物中心建成使用。全力办好“哈洽会”、“冰雪节”等品牌展会和节庆活动，拓展长江路会展经济带，加快建设农业国际博览中心。大力发展股权投资，设立创业投资引导基金，吸引更多的投资基金落户我市。继续做好哈尔滨银行等企业上市工作，支持符合条件的企业在资本市场挂牌融资。积极引进渣打、花旗等金融机构，确保法兴银行正式运营。二是深入实施产业发展创新驱动战略。着力构建政府搭台、院所创新、成果转化三位一体创新体系，继续抓好哈工大及高新技术企业科技成果转化平台、科技企业孵化器建设，科技应用技术类成果转化率保持在80%以上。强力推进高压无功发生器等30个重大高新技术产业化项目，支持企业牵头实施第三代核电技术等80项重大产业科技攻关计划，确保高新技术产业产值增长20%以上。三是努力提升园区产业承载集聚功能。坚持产业规划引领、基础设施先行、大项目支撑原则，完善重点园区控详规划和功能区规划，完成规划环境影响评价，为产业大招商、项目大建设创造条件。政府引导性资金提高到5亿元，支持重点园区加快基础设施建设，国家级开发区新开发面积都要超过5平方公里，26个重点园区产业集中度达到55%以上。经开区重点启动建设中国移动哈尔滨数据中心等大项目100个，新签约入驻项目100个；高新区重点推进光宇等15个科技产业项目建设，确保奥瑞德蓝宝石等50个项目竣工投产；利民开发区重点抓好哈药集团药物研发中心等30个项目建设，新引进企业30家；宾西开发区重点推进金红叶纸业等20个亿元以上项目建设，确保10个项目竣工投产。力争通过我们的切实努力，真正把国家级开发区打造成牵动哈大齐工业走廊发展的重要引擎。四是全力开展产业大招商。进一步创新招商举措，组建部门招商分局，主要领导负总责，抽调局级领导和后备干部，专职脱岗抓招商。区县（市）四大班子要人人担任务、个个扛指标，形成以区县（市）和开发区为主体、部门齐参与的产业招商大格局。积极改进招商方式，紧盯世界和国内500强、行业100强以及中央企业、品牌企业，全方位开展板块招商、产业链招商、以商招商，集中引进一批助推产业结构调整、发展方式转变和对俄经贸合作战略升级的大项目、好项目，全年新引进100亿元产业项目5个以上、50亿元11个以上、10亿元72个以上，实际使用外资增长20%，国内区域经济合作实际到位资金增长30%以上。产业建设是实现哈尔滨科学发展新跨越的动力所在、潜力所在、希望所在。我们一定要以对哈尔滨人民、对哈尔滨历史、对哈尔滨未来高度负责的政治胸怀和只争朝夕的进取精神，坚持大力度推动产业建设不动摇，不畏困难，不惧挑战，真抓实干，为全面建成小康社会奠定坚实的产业基础！

　　（二）持续壮大县域经济整体实力。深入实施富民强县战略，坚持以产业建设为支撑、以城乡一体化为牵动、以撤乡并村为抓手、以多渠道增加农民收入为目标，大力促进工业化和城镇化良性互动、城镇化和农业现代化相互协调，为县域经济更好更快发展注入强大的推动力。一是全力推进县域工业化。按照县域工业增加值和地方公共财政预算收入三年翻番，两年引进产业项目1000个、完成投资1000亿元的目标，全力推进产业强县工程，重点发展食品制造、农副产品加工等8大产业，建设乳制品、畜牧产品、禽类产品3大特色养殖加工板块，打造双城食品等10个加工基地，壮大宾西牛业等50户龙头企业，积极推进“巴木通方”区域经济一体化，努力形成优势互补、各具特色的县域产业发展新格局。二是加快推进农业现代化。把推进水利化、科技化、机械化、合作化、产业化、市场化作为发展现代化大农业的重要任务和主攻方向，突出抓好粮食产能稳定增长工程，粮食总产稳定在320亿斤以上。积极推进粮牧主辅换位，新建扩建规模化牧业养殖场（区）200个，建设村级畜牧兽医区域服务中心50个。继续实施“粮改菜”工程，重点加快双城、五常设施蔬菜基地建设。大兴农田水利，搞好中小河流治理和病险水库除险加固。实施农业科技创新工程，新建现代农业示范园区91个。加快发展农民专业合作组织，培育新型农业经营主体和新型职业农民，引导土地有序流转，发展适度规模经营，进一步激发农业生产活力。三是积极稳妥推进城镇化。坚持走集约、智能、绿色、低碳的新型城镇化道路，有序推动人口向城镇集中、土地向合作社集中、产业向园区集中。调整修编县（市）总体规划和中心镇规划。深入实施“百镇提档升级、二十镇试点引领”工程，抓好尚志、方正、通河次中心城市建设，以及南岗、香坊整区推进、每县（市）各1个整乡（镇）推进试点工作，集中打造团结镇、成高子镇等10个样板镇，搞好亚布力镇、依兰镇、拉林镇等一批城关镇、中心镇建设，着力提高城镇化质量。加快撤乡并村步伐，全年撤并乡（镇）14个、村（屯）268个，力争两年内城乡结合部乡（镇）一步跨入城市化。积极开展中心村建设，启动建设市级新农村示范村50个，推进扶贫开发项目400个，着力建设一批具有浓郁地方特色的美丽乡村。启动木兰松花江大桥建设和莫延公路前期工作，新建农村公路500公里、交工通车300公里，改造危险桥梁162座，着力打造主城区与县域“1小时经济圈”。县域经济是支撑全市产业建设的大舞台、破解“三农”问题的主阵地。全面建成小康社会关键在县域经济、难点在县域经济、希望也在县域经济。我们要以更大的力度和更有效的措施，让县域经济在未来加快发展中迸发出更大的活力！

　　（三）全面提升城市形象品质。坚持以建设区域性现代化国际化大城市为引领，高标准、高起点规划建设管理好城市，增强城市环境的吸引力和竞争力。一是搞好城市规划设计。严格依法执行城乡规划，进一步完善城市总体规划，确保城市空间布局、产业发展、基础设施、新老城区建设规划有效衔接。科学规划沿江景观生态旅游、机场路空港、松北大道商务办公等5条经济带，精心设计博物馆、哈尔滨火车站等5个重要节点，保护复兴道里、道外、南岗特色历史街区，展现城市疏朗大气的地域特征和欧陆风情的独特魅力。二是完善城市载体功能。顺应新战略纵深发展需求，持续加大城市建设投入力度，新建改造道路100条、桥涵20座，延伸打通红旗大街、职工街等一批主干街路，一苗圃高架桥、蓝天地道桥竣工通车。启动建设滨北公铁两用桥，改造绥满公路阿城绕行线等城市出入口。全面完成哈西地铁联络线主体工程，确保地铁1号线载客运营，启动建设1号线三期和2号线。提升静态交通承载功能，新建停车场20处，新增公共停车泊位1.2万个。加强综合交通枢纽建设，规划城市轻轨体系，支持哈佳、哈牡、哈尔滨至漠河快速铁路、太平国际机场扩建等重大项目建设。全力建设“公交都市”，辟建松花江水上公交航线和两岸旅游观光公交线路，新增、更新大容量公交车600台，新改扩建公交首末站30个，哈南综合交通枢纽和民生尚都等大型公交换乘枢纽竣工使用，努力实现市民便捷、温暖、安全出行。完善城市源网地理信息系统，加快推进供水、排水、供热、电力、燃气“五网并建”，重点实施先锋泵站改扩建、哈热六期、太平供热厂等项目建设，新建改造各类管网1000公里。三是提高城市管理水平。突出区街主体作用，积极落实长效机制，确保城市管理高效有序运行。实施精细化、智能化管理，持续提高机械化作业水平，城区机械化清扫率和清雪率分别提高到60%和70%，中心城区垃圾收运体系实现全覆盖，“数字城管”升级改造全面完成。深入推进城市环境综合整治，全力整治城乡结合部卫生环境及市场摊区、露天烧烤、基建工地、残土车等卫生污染源，努力让百姓生活的城市更干净、更整洁；全力整治违章建筑、随意扒设门窗、临街踏步、架空线缆、牌匾广告及屋顶雨搭，努力让百姓生活的城市更规范、更有序；全力整治乱停乱靠、非法营运、违章驾驶等交通顽疾，从严处罚、从严管理，改造完善现有交通信号设备，努力让百姓生活的城市更文明、更顺畅；全力整治老旧社区环境，开展“物业管理年”活动，创新物业管理体制机制，维修老旧住宅400万平方米，创建市级以上示范物业小区230个，努力让百姓生活的城市更宜居、更舒适。四是推进新区融合发展。突出生态、现代、大气、疏朗、清新定位，着力打造产业特色鲜明、功能布局合理、配套设施完善的现代化新城区。合理划分城市新区、行政区管理职责。统筹推进区域道路交通、源网和公共服务设施建设，不断增强新区人口集聚力和产业吸引力。加快推进哈西群力联络空间区域改造，打通龙葵路、龙腾路等15条区域道路，撬动和引领城市西部地区融合发展。启动哈东新区建设，推动城市东部地区加快振兴。为人民建设好城市、管理好城市、运行好城市，是政府义不容辞的责任。我们一定要科学规划、精细管理、全面建设，使哈尔滨不断朝着现代化国际化大城市目标迈进！

　　（四）扎实推进生态文明建设。牢固树立绿色发展理念，把保护环境生态和节能减排行动，贯穿到结构调整全过程和经济社会生活各方面，努力建设美丽生态宜居的哈尔滨。一是切实加强生态修复。突出打造“万顷松江湿地、百里生态长廊”，修订编制生态城市建设发展总体规划，抓好国家级水生态系统保护与修复试点工作，确保松花江流域年度治污任务全面完成，北国水城一期全线贯通，“三沟”年内实现清水入河，阿什河两年全部完成整治改造任务，充分展示滨水城市大气秀美、清爽宜人新形象。完成农村生态连片治理项目30个，治理水土流失36万亩。坚定不移地走“生态化、景观化、特色化”之路，着力打造森林城市，全年造林11万亩，城区新植树木50万株、新增绿地190公顷。二是切实加强环境保护。深入开展国家环保模范城创建活动，重点启动新华等4个污水处理厂建设，确保玉泉镇等5个污水处理厂实现通水调试，向阳垃圾厂污水处理升级改造和双琦垃圾处理改建工程全面完成，餐厨废弃物处理项目实现试运行，西南部生活垃圾处理场按期关闭，江北区域固体废弃物综合处理园区开工建设，污泥处置项目建成使用。持续加大市区分散燃煤小锅炉拆并改造力度，新增集中供热能力2200万平方米。定期发布PM2.5等环境质量监测数据。加强磨盘山等重点饮用水源地保护工作，确保人民群众饮水安全。三是切实加强节能减排。重点淘汰落后产能，水、气主要污染物排放总量比2010年分别削减3.2%和0.4%，完成既有建筑节能改造300万平方米。大力发展循环经济和环保产业，加快推进哈尔滨国家环保科技产业园等项目建设，不断提升经济社会可持续发展能力。我们要动员全社会力量，珍惜资源、保护环境，共同建设天蓝、地绿、水净美好家园，努力让哈尔滨走向生态文明新时代！

　　（五）继续深化改革扩大开放。坚持以改革增活力、以开放拓空间，着力在创新体制机制中破解发展难题，在扩大对外合作中寻求发展机遇，努力探索科学发展、跨越赶超的新途径。一是继续深化重点领域改革。进一步加快国资监管体制和国企产权制度改革，组建国有资产投资控股公司、交通集团和文旅集团，全面提升哈投、城投、水投、市政、地铁、物业供热等集团运营水平，构建“大国资”监管格局。改革公共财力配置方式，集中财力办大事，建立健全财政专项资金统筹安排、规范管理和绩效评价机制。稳步推进工资收入分配制度、农村产权制度、事业单位分类等改革。妥善处理政府与市场关系，以更大的勇气和智慧推动改革不断深入，坚决破除一切妨碍科学发展的既得利益障碍和体制机制弊端，最大限度解放和发展生产力，增强经济发展内生动力。二是积极促进对外经贸合作。在巩固美、韩等传统市场，开拓中东、拉美、非洲等新兴市场的同时，重点按照全省建设东北亚经济贸易开发区和哈牡绥东对俄贸易加工区的战略部署，加快构建对俄航空物流和陆海联运大通道，着力打造俄罗斯大宗产品交易、出口加工配送、经贸合作交流中心和会展、金融、信息服务平台，大力推进莫斯科莲花城国际贸易批发、诺林化工物流等项目建设，确保对俄进出口贸易总额增长50%以上。积极实施走出去战略，鼓励企业对外直接投资、扩大对外工程承包和劳务合作。全力做好综合保税区申报工作，力争早日开工建设。三是鼓励支持非公有制经济加快发展。营造更加宽松的政策环境，鼓励民间资本进入基础设施、公用事业、社会事业等领域。继续实施中小企业成长工程，完善中小企业服务大厅和中小企业服务平台，为企业提供“一站式”服务。充分发挥中小企业发展专项资金、政策性信用担保资金作用，促进中小微企业特别是科技型小微企业更好更快发展。我们要通过持续不断地深化改革、扩大开放，努力让哈尔滨发展动力更强劲、发展空间更广阔！

　　（六）着力保障和改善民生。牢记全心全意为人民服务宗旨，集中解决人民群众最关心、最直接、最现实的利益问题，努力使发展成果更多更公平惠及广大人民。今年重点实施10个方面、70个惠民行动项目，完成投资260亿元。一是努力办好人民满意教育。大力促进教育公平，合理配置教育资源，改造中小学校、配建新区义务教育学校、创建义务教育标准化学校、建立素质教育特色示范学校85所，新改扩建公办幼儿园80所，进城务工人员子女全部就读公办学校，让每个孩子都平等接受教育、成为有用之才。筹建哈尔滨体育职业技术学院，推进哈尔滨职业教育园建设，促进高等教育、继续教育、特殊教育和民族教育等各类教育协调发展。二是推动实现更高质量就业。牢牢把握民生之本，实施更加积极的就业政策，开展高层次人才、自主择业转业军官、高校毕业生、失业人员和农民等群体创业就业工程，精心办好哈尔滨创业博览会。全年新增城镇就业11.6万人，转移农村劳动力140万人。完善人才相关政策，把人才发展资金提高到1亿元，吸引更多的优秀人才创业哈尔滨、贡献哈尔滨。三是改善城乡居民住房条件。开工建设保障房2.5万套、竣工3.2万套，民生尚都、征跃小区、陶瓷小区等保障性安居工程建成入住，为1.3万户低收入住房困难家庭提供廉租房保障，发放经济适用房货币补贴4000万元。改造农村泥草房6.5万户、370万平方米，让城乡中低收入群体住房条件得到更大改善。四是健全社会保障体系。坚持全覆盖、保基本、多层次、可持续方针，继续扩大社会养老保险覆盖面，实现被征地农转非人员、机关事业单位及企业长期临时用工人员应保尽保。提高工伤保险参保率和失业人员待遇标准。建立城镇居民医保大病保险制度，报销比例不低于50%。适时启动城乡低保标准动态调整机制。完善社会养老服务体系，升级改造市属福利院，鼓励社会力量兴办养老机构。抓好“菜篮子”、“米袋子”工程，确保市场供应充足、物价基本稳定。落实供热、供水、交通等居民生活价格补贴政策，确保低收入群体基本生活不受大的影响。五是提高人民健康水平。扎实推进医药卫生体制改革，新农合政策内住院费用报销比例提高到75%，对17类37个病种实施重大疾病保障补偿。完善医疗卫生服务体系，实现城乡居民基本公共卫生服务全覆盖。强化食品药品安全监管。继续做好人口和计划生育工作，人口自然增长率控制在5％。以内。六是加快发展文化体育产业和事业。积极探索文化与科技、旅游、金融等融合发展新模式，大力培育新型文化业态。抓好大剧院、音乐厅、文化大世界等项目建设及运营管理，进一步提升国家和省级文化产业园规模效益，切实增强文化产业发展的牵动力。积极做好“731”旧址、“金上京”遗址申报世界文化遗产工作。全面发展群众体育和竞技体育，启动八区体育中心综合改造，打造城区“15分钟健身圈”，为丰富群众文体生活、提升城市软实力创造更多现实条件。七是加强和创新社会管理。深入实施“强基工程”，创新社区综合服务管理模式，新建千米以上中心社区、农村示范社区、创建四星级以上和谐社区105个。围绕打造全国最具安全感省会城市，继续深化“天网”工程，建设市应急联动指挥中心和公安交通指挥中心，依法打击各类违法犯罪。加强公共安全管理，有效遏制道路交通、火灾、生产等重大恶性事故。落实重大决策社会稳定风险评估和社会矛盾纠纷排查机制，完善大调解工作体系，着力化解多年积累的群体性突出矛盾，努力创造和谐稳定的社会环境。八是持续推进精神文明建设。围绕构建社会主义核心价值体系，加强社会公德、职业道德、家庭美德和个人品德教育。深入推进全省“三优”文明城市和全国文明城市创建工作，广泛开展志愿服务、学雷锋、学道德模范及文明创建评比等活动，努力塑造文明哈尔滨新形象。继续做好广播影视、新闻出版、民族宗教、妇女儿童、外事侨务、国防教育、双拥及民兵预备役等工作。

　　三、加强政府自身建设

　　面对跨越发展新要求和人民群众新期待，我们要以深入学习贯彻落实十八大精神为引领，认真落实改进工作作风、密切联系群众八项规定，努力打造职能科学、结构优化、廉洁高效、人民满意的服务型政府。

　　（一）进一步保持昂扬向上、奋发有为的精神状态。坚定发展信心。始终保持一种加快发展的“精气神”，不断增强敢为人先的锐气、不等不靠的志气、蓬勃向上的朝气，解放思想、创新实践，彻底打破陈旧观念和落后思想的桎梏，在体制创新、产业招商、大项目建设等重点工作上大胆地闯、大胆地试，知难而进不言难，迎难而上不怕难，充分展现“逢山开路、遇河架桥”的进取精神。牢记发展使命。始终带着感情、责任、追求开展工作，保持一种振兴哈尔滨锲而不舍、永不言败的执着精神，用勇气去拼搏、用魄力去开拓、用智慧去创造，在哈尔滨率先全面建成小康社会的新征程中创一流、当排头。拓宽发展视野。善于把哈尔滨放在全省全国乃至世界发展的大格局中去分析、去衡量，谋划工作不急功近利，推动工作不心浮气躁，以更加宽广的视野审视自身发展，以更加开放的思维谋划区域经济，以更加积极的措施推进改革开放。

　　（二）进一步倡导雷厉风行、真抓实干的工作作风。端正抓落实的态度。在其位、谋其政、尽其责，始终保持一种孜孜以求、一丝不苟的精神，保持一种干就干好、精益求精的韧劲，保持一种大刀阔斧、披荆斩棘的勇气，对认定的事抓住不放、不优柔寡断，对定下的事持之以恒、不瞻前顾后，对已干的事一抓到底、不半途而废。提高抓落实的能力。着重在公务员队伍中营造崇尚学习的良好风气，少应酬多学习，少休闲多思考，少空谈多干事，找准发展症结，把握政策机遇，增强工作落实的前瞻性、主动性和针对性。强化抓落实的责任。对园区建设、征地拆迁、源网管理、撤乡并镇等难点任务，敢于担当、敢于承重、敢于碰硬、敢于超越自我，努力营造只为落实想办法、不为落后找理由的工作氛围。增强抓落实的合力。认真接受人大代表的依法监督和政协委员的民主监督，充分发挥工会、共青团、妇联等人民团体桥梁纽带作用，对产业建设、城市发展、民生改善等事关全局和长远的大事要事，始终做到目标同向、工作同频、行动同步，在更大范围更广领域构筑推动发展的强大合力。

　　（三）进一步营造服务产业、促进发展的经济环境。强化行政问责。严格落实行政问责和整治涉企“四乱”规定，及时对破坏经济发展环境的人和事进行严厉的查处，促使广大政府工作人员尽职尽责、尽心尽力，真正把心思用在谋发展上、把精力放在抓落实上、把功夫下在见成效上。强化服务质量。尊重企业、尊重创造、尊重群众的首创精神，坚持多服务少干预、多支持少指责、多协调少扯皮，切实帮助企业解决发展难题；继续深化行政审批制度改革，简政放权，优化流程，努力为企业提供更周到、更具体、更优质的服务；切实发挥“纠风直通车”、效能监察中心、市长公开电话作用，畅通诉求渠道，努力让企业和群众的合法权益得到充分保障。强化绩效管理。建立健全绩效评估制度体系，把绩效管理手段体现在政府工作的全过程，切实提高政府行政效能，增强政府工作的执行力和创造力。

　　（四）进一步树立清正廉洁、干净干事的良好形象。加强廉政体系建设。坚持以制约和监督权力运行为核心，构建权责清晰、流程规范、制度管用、预警及时、防控有效的廉政风险防控管理机制。加强廉洁自律教育。牢固树立正确的权力观、利益观和政绩观，自觉遵守廉政准则，老老实实做人、干干净净做事。加强廉政制度建设。注重用制度管权管事管人，切实加大对领导干部和国企领导人经济责任审计力度，从源头上预防腐败行为发生。加强廉政社会监督。切实增强政府决策、政务信息、行政过程和行政程序透明度，让权力在阳光下运行。

　　各位代表，建设繁荣幸福文明和谐的哈尔滨，责任重大、使命光荣。让我们紧密团结在以习近平同志为总书记的党中央周围，在省委、省政府和市委领导下，紧紧依靠全市人民，凝神聚力谋发展、千方百计惠民生，为实现哈尔滨科学发展新跨越、谱写率先全面建成小康社会新篇章而努力奋斗！
